# HIV-1 Nef increases astrocyte sensitivity towards exogenous hydrogen peroxide

**DOI:** 10.1186/1743-422X-8-35

**Published:** 2011-01-22

**Authors:** Sabine Masanetz, Michael H Lehmann

**Affiliations:** 1 Institute of Virology, Technical University of Munich/Helmholtz Zentrum München, 81675 Munich, Germany; 2 Institute for Infectious Diseases and Zoonoses, Ludwig-Maximilians-University Munich, 80539 Munich, Germany

## Abstract

**Background:**

HIV-1 infected individuals are under chronic exposure to reactive oxygen species (ROS) considered to be instrumental in the progression of AIDS and the development of HIV-1 associated dementia (HAD). Astrocytes support neuronal function and protect them against cytotoxic substances including ROS. The protein HIV-1 Nef, a progression factor in AIDS pathology is abundantly expressed in astrocytes in patients with HAD, and thus may influence its functions.

**Results:**

Endogenous expressed HIV-1 Nef leads to increased sensitivity of human astrocytes towards exogenous hydrogen peroxide but not towards TNF-alpha. Cell death of *nef*-expressing astrocytes exposed to 10 μM hydrogen peroxide for 30 min occurred within 4 h.

**Conclusion:**

HIV-1 Nef may contribute to neuronal dysfunction and the development of HAD by causing death of astrocytes through decreasing their tolerance for hydrogen peroxide.

## Background

Both HIV-1 associated dementia (HAD) and a milder form of HIV-1 associated cognitive disorder, known as minor cognitive and motor disorder (MCMD) are frequent complications of the acquired immunodeficiency syndrome (AIDS) and are characterized by neuronal dysfunction and cell death caused by HIV-1 through direct and indirect mechanisms [1-4].

Recently, a sophisticated inspection of brains from HIV-1 infected patients confirmed that neurons are not infected with HIV-1 and surprisingly revealed that astrocytes, the most abundant cell type in the brain, are extensively infected. Additionally, this study elucidated that infection of astrocytes with HIV-1 correlated with the severity of neuropathology [5]. Astrocytes play an important role in maintaining homeostasis, providing neuroprotection and regulating physiological activities within the brain [6-8]. Therefore, astrogliosis and astrocyte death occurring in HIV-infected individuals [9-12] may contribute indirectly to neuronal dysfunction.

Even though HIV-1 is integrated in the astrocyte genome, it rarely replicates in this cell type *in vivo*, however, regulatory proteins such as Nef are found to be abundantly expressed [13-15]. The presence of HIV-1 Nef in the brain is associated with astrogliosis and recruitment of monocytes/macrophages [16], correlating with the development of HAD [17].

Astrocytes stably transfected with HIV-1 Nef function as appropriate cellular model systems for the purpose of investigating basic mechanisms pertinent to the understanding of HAD pathogenesis. Using these cells, we previously showed that HIV-1 Nef induces CCL2/MCP-1 [18], thereby, providing an alternative hypothesis for the occurrence of this chemokine at high concentrations in the cerebrospinal fluid (CSF) of HIV-infected individuals with HAD [19,20]. CCL2 plays an important role in the cerebral infiltration of monocytes/macrophages in these patients [21,22]. Infiltrated and activated monocytes/macrophages, which are considered to be the effector cells in cellular and tissue damage in AIDS, produce cytotoxic substances such as reactive oxygen species (ROS) and inflammatory cytokines [23,24]. Consequently, HIV-1 infected and non-infected astrocytes are subjected to an environment characterized, amongst others, by high concentrations of hydrogen peroxide and tumor necrosis factor (TNF)-alpha. Therefore, the aim of this study was to investigate the effect of HIV-1 Nef on the cellular viability of human astrocytes exposed to these particular cytotoxic substances.

## Results

### Astrocytes stably transfected with *HIV-1 nef *are highly sensitive to hydrogen peroxide induced cell death

**Figure 1:**
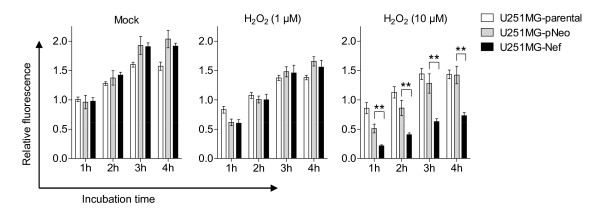
**Hydrogen peroxide decreased the viability of *nef*-expressing astrocytes**. U251MG-parental, -pNeo and -Nef cells were treated with hydrogen peroxide for 30 min at indicated concentrations. Subsequently cells were washed twice with PBS and incubated in VLE-RPMI 1640 medium containing 10% FCS for a further 24 h. The medium was then exchanged and cell viability assay was performed as described in the Material section. The times indicated are relative to the moment of adding AlamarBlue^® ^reagent to the cell culture medium. The relative fluorescence represents the ratio of the fluorescence intensity of study cells versus mock-treated cells at 1 h after start of the assay. Data represent mean ± s.e.m. (n = 6); **, P < 0.01.

Astrocytes fulfil a protective function for neurons through elimination of ROS such as hydrogen peroxide [25]. Yet astrocytes are more vulnerable to the effects of hydrogen peroxide than neurons [26,27], but it is not known how this is modulated by HIV-1 Nef. Therefore, the sensitivity of human astrocytic U251MG-Nef cells towards hydrogen peroxide was tested in comparison with the sensitivity of U251MG-parental and U251MG-pNeo cells. Cells treated with hydrogen peroxide at concentrations of 1 μM and 10 μM for 30 min were investigated after 24 h for viability using AlamarBlue^® ^reagent containing resazurin, a non-toxic, oxidation-reduction indicator indicating mitochondrial metabolic activity. The analysis revealed that in astrocytic cells stably expressing *nef*, hydrogen peroxide significantly reduced the cell viability as compared to mock-treated cells, hydrogen peroxide-treated U251MG-parental cells and hydrogen peroxide-treated U251MG-pNeo cells (Figure 1). Similar results were obtained at 48 h (additional file 1).

### Hydrogen peroxide rapidly induced cell death of astrocytes stably transfected with *HIV-1 nef*

**Figure 2:**
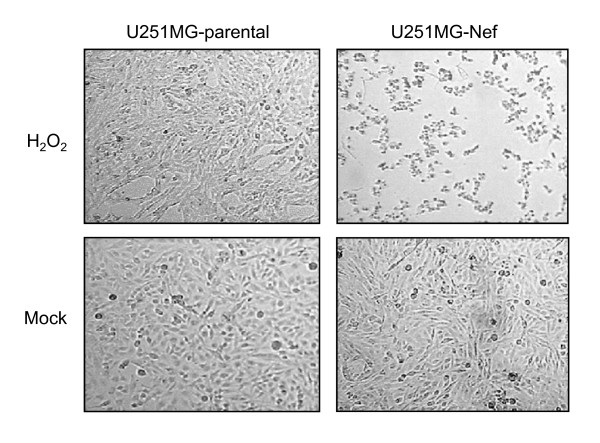
**Hydrogen peroxide leads to rapid detachment of *nef*-expressing astrocytes**. U251MG-parental and -Nef cells were treated with hydrogen peroxide (10 μM) for 30 min. Cells were subsequently washed twice with PBS, incubated in VLE-RPMI 1640 medium containing 10% FCS for a further 3 h 30 min and subsequently a light microscopic analysis of astrocytic cells was performed. A Zeiss Axiovert 25 microscope (Carl Zeiss Jena GmbH, Jena, Germany) was used. Original magnification, × 100.

A light microscopic analysis was performed in order to examine whether signs of cell death induced by hydrogen peroxide may be detected earlier than 24 h in *nef*-expressing astrocytes. Indeed, in contrast to the control cells, the previously flat-shaped *nef*-expressing astrocytes had undergone a morphological alteration to being round-shaped and almost completely detached from the cell culture flask surface 3 h 30 min subsequent to 30 min treatment with hydrogen peroxide at a concentration of 10 μM (Figure 2).

**Figure 3:**
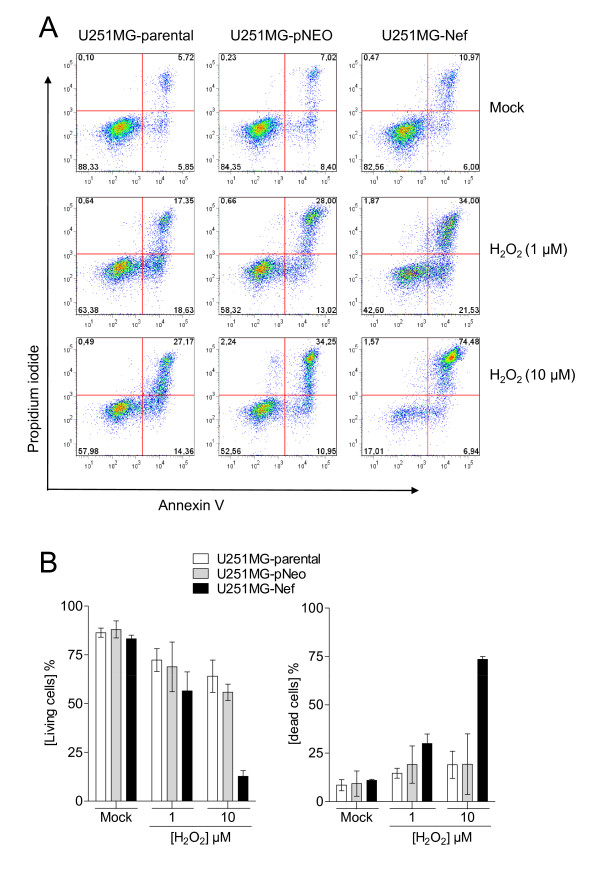
**Hydrogen peroxide leads to rapid loss of the cell membrane integrity in *nef*-expressing astrocytes**. (A) U251MG-parental, -pNeo and -Nef cells were treated with hydrogen peroxide for 30 min at concentrations as indicated. Cells then were washed twice with PBS, incubated in VLE-RPMI 1640 medium containing 10% FCS for a further 3 h 30 min and subsequently the annexin V assay was performed as described in the Methods section. (B) Summary of three independent experiments. Annexin V, PI double-negative cells (living cells) and annexin V, PI double-positive cells (dead cells) are shown.

Translocation of the membrane phospholipid phosphatidylserine (PS) to the outer leaflet of the plasma membrane occurs rapidly after exposure to a cytotoxic agent and mostly indicates a point-of-no-return during the cellular dying process. Using the Annexin V assay, it was confirmed that hydrogen peroxide at a concentration of 10 μM severely affected cellular viability of U251MG-Nef cells but had only a small effect on astrocytic U251MG-parental and U251MG-pNeo cells. PS exposure on the cell surface in combination with positive PI staining indicating loss of plasma membrane integrity, which is criterion to consider a cell as dead [28], has been detected in about 75% of the *nef*-expressing cells (Figure 3).

### Astrocytes stably transfected with *HIV-1 nef *are as sensitive to TNF-alpha induced cell death as non-transfected cells

**Figure 4:**
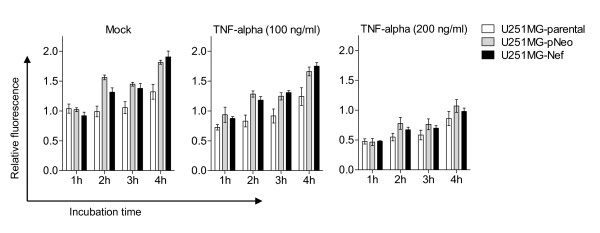
**HIV-1 Nef does not modulate TNF-alpha decreased viability of astrocytes**. U251MG-parental, -pNeo and -Nef cells were treated with TNF-alpha for 24 h at indicated concentrations. The medium was then exchanged and cell viability assay was performed as described in the Material section. The times indicated are relative to the moment of adding AlamarBlue^® ^reagent to the cell culture medium. The relative fluorescence represents the ratio of the fluorescence intensity of study cells versus mock-treated cells at 1 h after start of the assay. Data represent mean ± s.e.m. (*n *= 5).

Previously, it has been shown that HIV-1 Nef protects T cells against TNF-alpha induced apoptosis [29]. Consequently, we tested whether HIV-1 Nef is also capable of protecting astrocytes against TNF-alpha induced cell death. Human astrocytic U251MG-parental, -pNeo and -Nef cells were treated with TNF-alpha for 24 h and their viability was analysed using AlamarBlue^® ^reagent. Data revealed that TNF-alpha significantly reduced the cell viability of each astrocytic cell type investigated here to a similar degree including the stably *nef*-transfected cells (Figure 4). This result has been confirmed using the Annexin V assay (additional file 2).

## Discussion

Chronic oxidative stress in HIV-infected patients plays an important role in AIDS progression [30,31]. This phenomenon is explained by a depletion of endogenous antioxidant moieties and an increased production of ROS. Oxidative stress, in particular, is thought to be a cause of neuronal cell death in the brain of HIV-1 infected patients and believed to contribute to development of HAD [32,33]. Moreover, ROS-induced astrocyte death is also thought to play a role in the occurrence of HAD [26,27].

Here we show that a short exposure of exogenous hydrogen peroxide to *nef*-expressing astrocytes led to their rapid cell death. The early detection of a high number of propidium iodide/annexin V double positive cells points to necrotic cell death [34], which was previously suggested when astrocytes are subjected to tertiary-butyl hydroperoxide [35]. But it can not be finally defined only from this observation what kind of cell death exactly occurred in our model. Also it depends on the concentration of hydrogen peroxide applied whether a cell dies in an apoptotic or necrotic manner [36]. In this context it is interesting to note that astrocytes are vulnerable to hydrogen peroxide at concentrations ranging from 0.5 mM to 2.5 mM [27], values approximately a 1.000 fold higher than the concentration applied to induce death of *nef*-expressing astrocytes herein. So it remains a challenge for further studies to elucidate what HIV-1 Nef precisely alters in the cell leading to increased sensitivity to exogenous hydrogen peroxide. Intriguingly, it has been shown during the preparation of this manuscript that HIV-1 Nef in primary human astrocytes and in the brain of mice increases oxidative stress [37], which is in line with our finding.

Since HIV-1 Nef is known to inhibit apoptosis of T-cells [29,38,39] and monocytes/macrophages [40,41], it was somewhat surprising that TNF-alpha decreased the viability of U251MG-Nef cells and U251MG-parental cells equally. Additionally, this finding is in contrast to previously reported data demonstrating that HIV-1 Nef prevents TNF-alpha triggered apoptosis in astrocytic U251MG cells [42]. This discrepancy may be due to the use of cells stably transfected with *nef *in our study, which could clearly well simulate the long term effect of HIV-1 Nef in chronically infected cells [43] than cells transiently transfected with *nef*. Moreover, involvement of HIV-1 Nef in cell survival is subject to generally controversy [44,45].

HIV-1 encodes a glutathione peroxidase [46], which has been shown to protect the cell against exogenous and endogenous ROS [47]. Consequently, what ever the reason why HIV-1 Nef causes an increase of sensitivity towards hydrogen peroxide, it is conceivable that the HIV-1 GPX could counteract this action of HIV-1 Nef by detoxifying hydrogen peroxide. Thereby HIV-1 GPX would prevent the cytotoxic potential of HIV-Nef, which is considered as a progression factor in AIDS [48-50] and known to induce an AIDS-like disease in a mouse model [51,52]. Thus, this could explain the paradoxical effect that functional HIV-1 GPXs are frequently found in long-term non-progressors while non-functional HIV-1 GPXs are present in HIV-1 isolates from patients developing AIDS [47].

## Conclusions

Besides other known direct and indirect effects of HIV-1 proteins, HIV-1 Nef may contribute to cellular and tissue injury frequently detected in HIV-1 infected individuals, including various AIDS-associated diseases such as HAD, by increasing the sensitivity of Nef-harboring cells to hydrogen peroxide.

## Methods

### Cell culture

**Figure 5:**
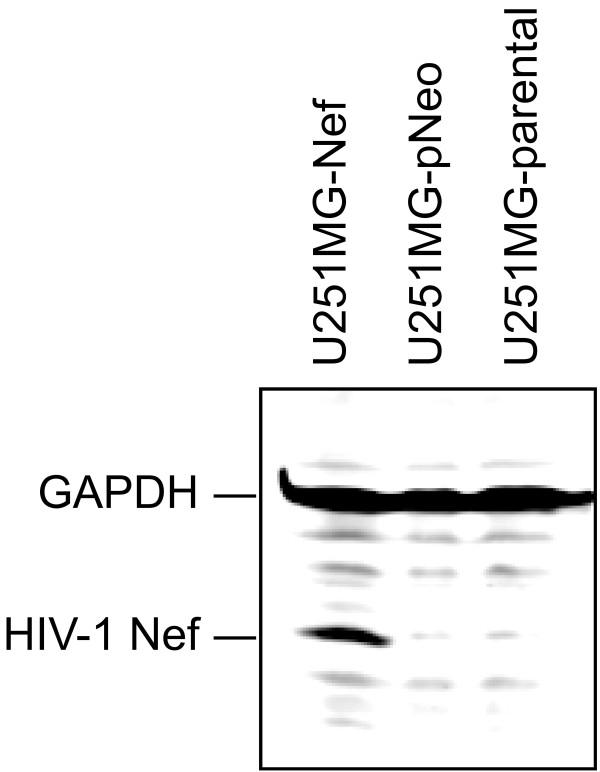
**Detection of HIV-1 Nef by immunoblotting**. Lysates of U251MG-parental, -pNeo and -Nef cells were prepared, separated and blotted, and HIV-1 Nef and GAPDH have been detected as described in the Methods section.

The human astrocytoma cell line U251MG was obtained from M. Brenner (National Institutes of Health, Bethesda, MD). The cell lines U251MG-Nef_Bru _clone 4/4.2 stably expressing *nef *from HIV-1_Bru _(GenBank accession number K02013) and U251MG-pNeo carrying only the neomycin resistance gene were established as reported [53]. HIV-1 Nef expression was confirmed by immunoblotting (Figure 5). Cells were routinely incubated at 37° under 5% CO2, and 90% humidity in VLE-RPMI 1640 medium certified to contain < 0.01 endotoxin units/ml, and supplemented with 10% fetal calf serum (FCS), 100 U/ml penicillin as well as 100 μg/ml streptomycin (all from Biochrom AG, Berlin, Germany). Before treatment with hydrogen peroxide (Merck KgaA) or TNF-alpha (BioSource International Inc., Camarillo, CA), cells were seeded at a density of 1 × 10^5 ^cells/ml in 96-well flat bottomed microtiter plates (BD Biosciences) for the cell viability assay or in 12-well plates (Costar) for the annexin V assay and incubated overnight in VLE-RPMI 1640 medium supplemented with 10% FCS.

## Immunoblotting and immunodetection

Lysates of U251MG-parental, -pNeo and -Nef cells were prepared by directly adding 1x SDS sample loading buffer to the cells followed by sonication. Samples were separated on a 4-20% tris-glycine gradient gel (Anamed, Darmstadt, Germany) and blotted on a nitrocellulose membrane. The blotted membranes were immunostained using mouse anti-Nef 3E6 mAb provided by K. Krohn through the National Institute for Biological Standards and Control Centralised Facility for AIDS Reagents, mouse anti-GAPDH mAb MAB347 (Chemicon International, Inc., Temecula, CA) and MFP488-conjugated goat anti-mouse antibody (MoBiTec GmbH, Göttingen, Germany), and positive signals were detected by fluorescence scanning (excitation wavelength 488 nm, emission filter 520BP40) using the Typhoon 9410 Fluorescence Scanner (GE Healthcare), and analyzed using ImageQuant 5.2 software (Molecular Dynamics).

## Cell viability assay

The AlamarBlue^® ^reagent (Molecular Probes, Inc., Eugene, OR) containing the water soluble, non-toxic dye resazurin (7-Hydroxy-3*H*-phenoxazin-3-one 10-oxide) was used to quantify mitochondrial activity according to the manufacturer's recommendation. Briefly, 1/10th of the volume of AlamarBlue^® ^reagent was added directly to the cells in culture medium. Using the Typhoon™ 9410 fluorescence scanner (GE Healthcare), fluorescence measurement was performed by applying an excitation wavelength of 532 nm and an emission filter of 580BP30 nm. Data were analyzed using ImageQuant™ TL software (GE Healthcare). The fluorescence intensity of medium containing only AlamarBlue^® ^was determined simultaneously and was subtracted from all values.

## Annexin V assay

Phosphatidylserine on the cell surface was detected with the Annexin V-FITC Apoptosis Detection Kit I (BD Biosciences). Briefly, cells were plated and treated in 12-well plates (Costar). Then cells were washed twice with cold PBS and incubated in the dark for 15 min in 1 × binding buffer supplemented with annexin V-FITC. Propidium iodide (PI) was added to the cell suspension immediately before analyzing with the BD FACSCanto™ flow cytometer (BD Biosciences). Data were evaluated using FlowJo© software (Tree Star).

## Statistical analysis

GraphPad Prism 4 (GraphPad Software, Inc., San Diego, CA) was used for statistical analysis. The Mann-Whitney test was used to compare the groups; a *P *value of less than 0.05 was considered significant. Tests were performed exactly and two-tailed.

## Competing interests

The authors declare that they have no competing interests.

## Authors' contributions

MHL conceived and designed the experiments, SM performed the experiments, SM and MHL analyzed the data, MHL wrote the paper. All authors have read and approved the final manuscript.

## Authors' information

After receiving her M.Sc. in Molecular Biotechnology, SM moved to the Physiology Weihenstephan, Technical University Munich, Freising, Germany to work for her PhD.

MHL received his PhD in Biology from the Friedrich-Schiller-University of Jena, Germany and currently holds a faculty position at the Institute for Infectious Diseases and Zoonoses, Ludwig-Maximilians-Universität München, Germany.

## Supplementary Material

Additional file 1 **Hydrogen peroxide significantly decreased the viability of *nef*-expressing astrocytes**. U251MG-parental and -Nef cells were treated with hydrogen peroxide for 30 min at indicated concentrations. Cells were subsequently washed twice with PBS, incubated in VLE-RPMI 1640 medium containing 10% FCS for a further 48 h. The medium was then exchanged and cell viability assay was performed as described in the Methods section. The relative fluorescence represents the ratio of the fluorescence intensity of study cells versus mock-treated. Data obtained after 4 h of starting the assay represent mean ± s.e.m. (n = 6); **, P < 0.01. Click here for file

Additional file 2 **TNF-alpha equally induces PS externalization in U251MG-parental and -Nef cells**. Cells were treated with TNF-alpha for 4 h or with hydrogen peroxide for 30 min at concentrations as indicated. Cells treated with hydrogen peroxide were washed twice with PBS, incubated in VLE-RPMI 1640 medium containing 10% FCS for a further 3 h 30 min and subsequently the annexin V assay was performed as described in the Methods section. Click here for file
