# Supplementary figures and images for: HIV-1 Nef increases astrocyte sensitivity towards exogenous hydrogen peroxide
Source: Virol J. 2011 Jan 22;8:35. doi: 10.1186/1743-422X-8-35 (PMC3038946; doi:10.1186/1743-422X-8-35)

## Slide 1
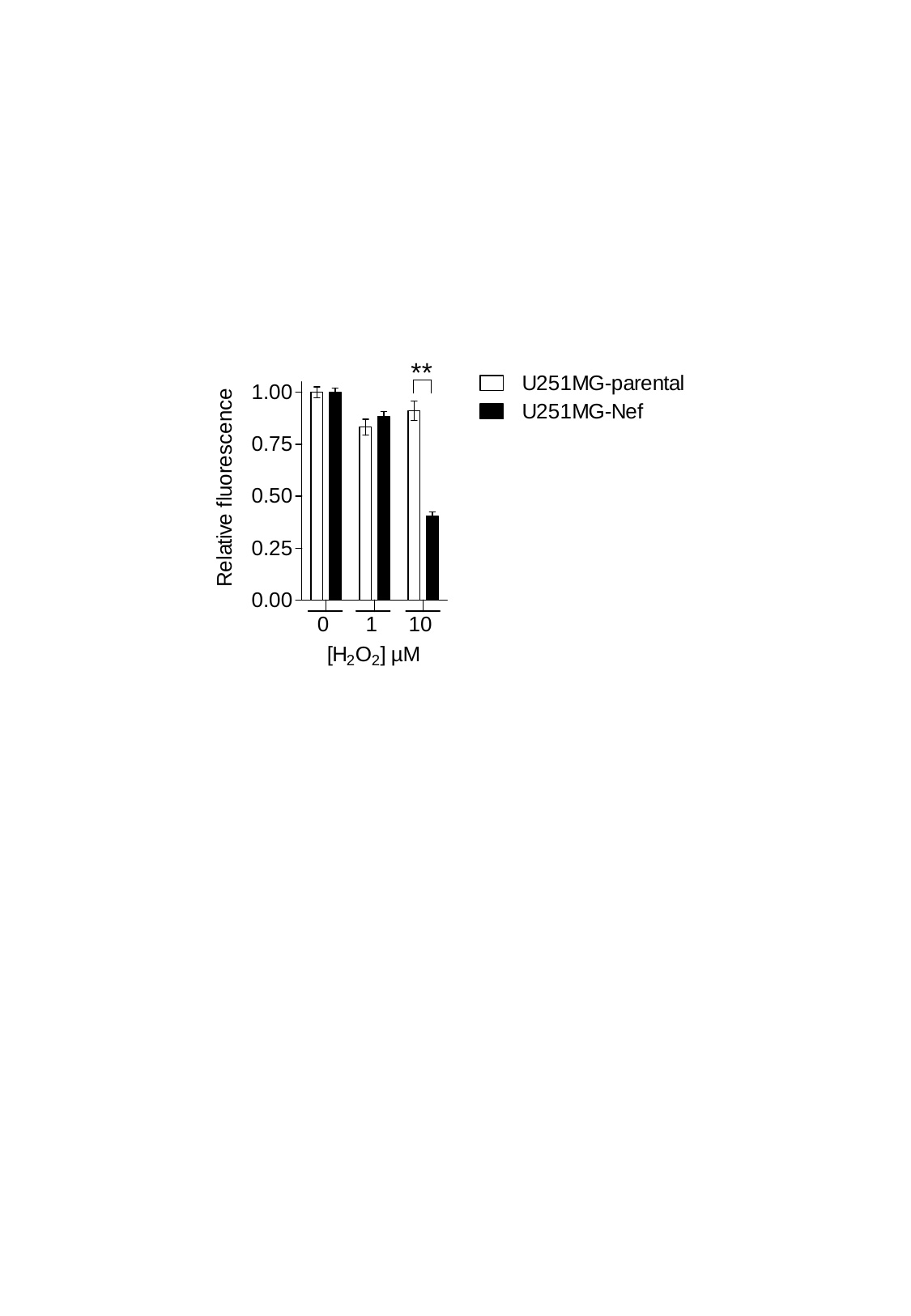

Supplement: Additional file 1 — Hydrogen peroxide significantly decreased the viability of nef-expressing astrocytes. U251MG-parental and -Nef cells were treated with hydrogen peroxide for 30 min at indicated concentrations. Cells were subsequently washed twice with PBS, incubated in VLE-RPMI 1640 medium containing 10% FCS for a further 48 h. The medium was then exchanged and cell viability assay was performed as described in the Methods section. The relative fluorescence represents the ratio of the fluorescence intensity of study cells versus mock-treated. Data obtained after 4 h of starting the assay represent mean ± s.e.m. (n = 6); **, P < 0.01. [file 1743-422X-8-35-S1.PPT]

## Slide 1
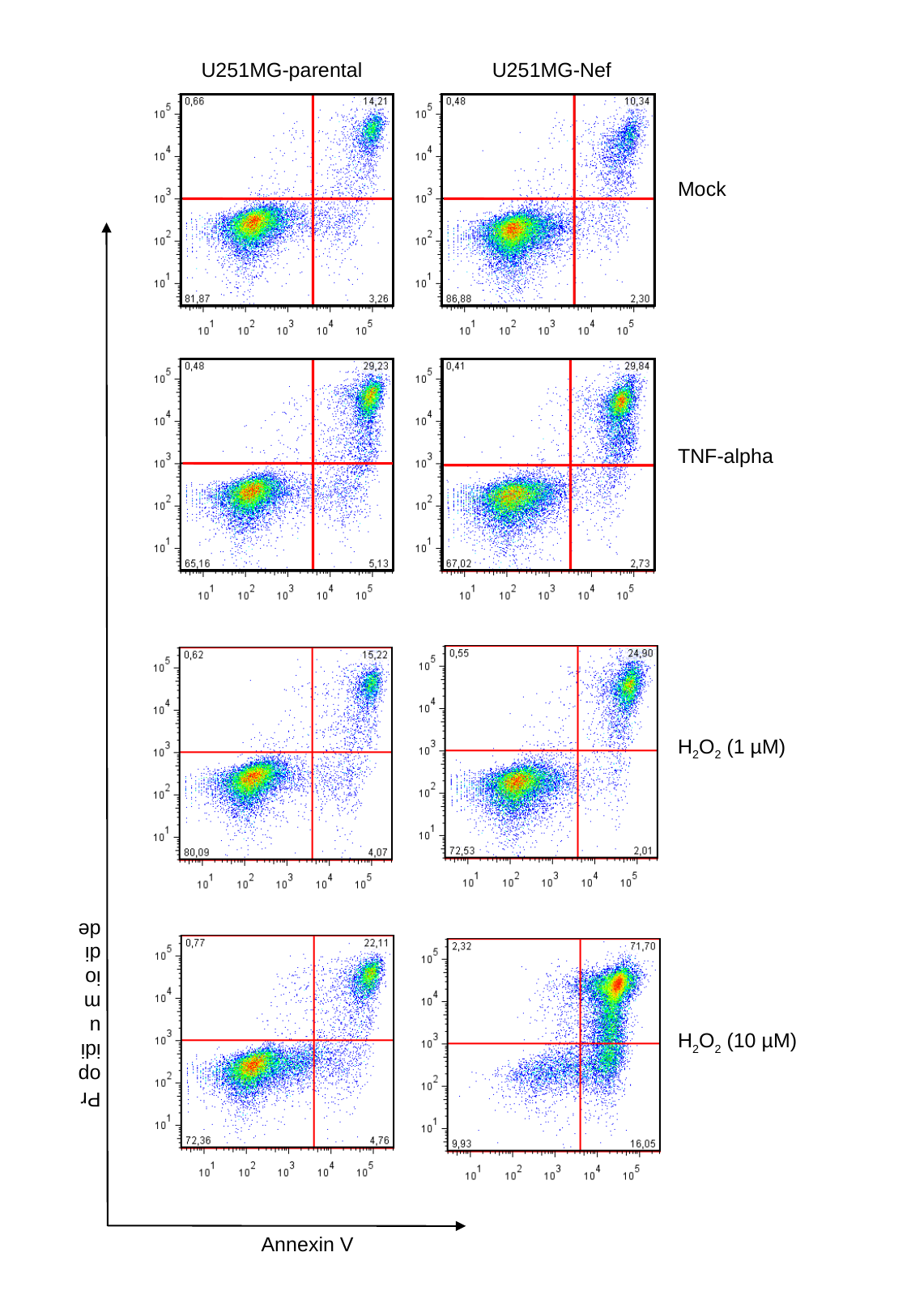

U251MG-parental U251MG-Nef
Mock
TNF-alpha
H2O2 (1 µM)
H2O2 (10 µM)
Propidium iodide
Annexin V

Supplement: Additional file 2 — TNF-alpha equally induces PS externalization in U251MG-parental and -Nef cells. Cells were treated with TNF-alpha for 4 h or with hydrogen peroxide for 30 min at concentrations as indicated. Cells treated with hydrogen peroxide were washed twice with PBS, incubated in VLE-RPMI 1640 medium containing 10% FCS for a further 3 h 30 min and subsequently the annexin V assay was performed as described in the Methods section. [file 1743-422X-8-35-S2.PPT]
